# Experiences of rheumatoid arthritis patients with digital health technologies in self-management: a qualitative study

**DOI:** 10.1007/s00296-026-06177-2

**Published:** 2026-06-05

**Authors:** Keriman Ayteki̇n Kanadlı, Nermin Olgun, Yasemin Sazak

**Affiliations:** 1 Department of Education, Hatay Mustafa Kemal University Hospital, Hatay, Turkey Antakya, 31000; 2 Department of Nursing, Faculty of Health Sciences, Hasan Kalyoncu University, Gaziantep, Turkey; 3 Department of Nursing, Faculty of Health Sciences, Kahramanmaraş Istiklal University, Kahramanmaraş, Turkey

**Keywords:** Rheumatoid arthritis, Digital health, Self-management, Chronic disease, Nurse, Qualitative research

## Abstract

Effective self-management in rheumatoid arthritis directly affects treatment, follow-up, and care processes. Individuals with chronic diseases can take an active role in their self-management by accessing healthcare professionals and information resources through digital health technologies. This study was conducted to examine the experiences of patients with rheumatoid arthritis in using digital health technologies in their self-management processes. This study was conducted in a university hospital in southern Türkiye with patients diagnosed with rheumatoid arthritis and employed a descriptive qualitative design. This study, data were collected using a “Patient Information Form” and a " Semi-Structured Interview Form”. Using purposive sampling, the study was completed with 25 participants. Data were evaluated using thematic analysis. The 32-item Consolidated Criteria Checklist (COREQ) was used in the reporting process of the research. The average age of the participants was 36.4 ± 10.4, and 80% were female. From the analysis of the data, three main themes and seven sub-themes were created: “use of digital health technologies”, “digital health technologies in self-management” and “expectations from digital health technologies”. This study found that patients with rheumatoid arthritis primarily use digital health technologies for accessing information, scheduling appointments, and supporting self-management in daily life. Furthermore, it was observed that patients have significant expectations regarding integrated digital health applications with healthcare professionals to ensure effective self-management. In this sense, it is recommended that digital tools be designed and experimental studies be conducted in this area, taking into account the expectations of patients with rheumatoid arthritis.

**Supplementary Information:**

The online version contains supplementary material available at 10.1007/s00296-026-06177-2.

## Introduction

Rheumatoid arthritis (RA) is a chronic, systemic, and inflammatory rheumatic disorder with an unknown etiology [1, 2]. As its prevalence grows with age, RA has been reported in approximately 0.5% to 1.0% of the adult population globally. This disease manifests itself with a feeling of overall discomfort, pain, severe fatigue and joint damage and progresses with exacerbations. These exacerbations have a serious negative impact on quality of life and require effective symptom management, including medical treatment [2, 3]. Despite recent advances in treatment, most of patients with RA have difficulty with their daily life activities [2]. In this sense, the development of self-management skills to overcome the difficulties of living with RA plays a decisive role in mitigating the effects and progression of the disease [4, 5]. Furthermore, the 2021 recommendations from the European Alliance of Associations for Rheumatism (EULAR) defined self-management as a holistic approach that empowers individuals with inflammatory arthritis to understand their disease, actively participate in the treatment process and make decisions together with healthcare professionals [5]. Accordingly, an effective self-management in RA positively affects treatment, follow-up, and care processes, as well as the quality of life, and raises the effectiveness of care [1].

Recently, rapid advances in digital technologies have led to an upsurge in the needs, digital demands, and expectations of individuals. The widespread use of both institutional and individual digitalisation has led to the restructuring of healthcare services, as in every sector [6]. The prevalence of chronic diseases, rising health expenditures, the needs that cannot be adequately met with available health services and the limited number of available healthcare professionals further grow the importance of digital health technologies (DHTs) [1, 6]. Individuals can take a more active role in their self-management by accessing healthcare professionals and reliable sources of information through DHTs [1, 6, 7]. DHTs are reported to be effective in monitoring chronic diseases and supporting self-management processes [8]. Similar studies have also reported that digital health-based nursing approaches contribute to both the improvement of clinical outcomes and the more efficient use of healthcare resources [7, 9]. It has also been found that DHTs support individuals with RA to take an active role in their disease management and to reduce the burden on the healthcare system [1]. Although the inclusion of DHTs into the care processes of disease management in RA has been increasing day by day, it has been observed that the literature mainly focuses on issues such as quantitative methods, the level of technology use or the effectiveness of specific digital tools [10–15]. However, qualitative evidence on how patients with RA use DHTs in their daily activities, the impact of these tools on their self-management processes and their expectations is limited [16–18]. No qualitative study was found that evaluates patients’ experiences from a self-management perspective, especially in the Turkish context. Accordingly, this study aims to present important findings on the development of patient-oriented digital tools and the strengthening of holistic self-management approaches in clinical and nursing care by examining the experiences of patients with RA in the use of DHTs.

## Method

### Design

This descriptive qualitative study [19] was conducted in a university hospital located in the southern region of Türkiye. The reporting process of the research was based on the 32-item Consolidated Criteria for Reporting Qualitative Research (COREQ) checklist [20].

### Research team

The research team included three authors. A 38-year-old KAK held a PhD degree and worked as a faculty member at the relevant hospital. A 61-year-old NO was a professor, and a 43-year-old YS was an assistant professor and lectured at the faculty of nursing. The researchers worked as nurses in the clinic in the past and were trained in qualitative research.

### Context of the research and participants

The participants consisted of individuals who attended routine controls at the rheumatology outpatient clinic of a university hospital in southern Türkiye. Data was collected between July 3, 2025 and August 22, 2025. The participants were individuals who were over 18 years old, could communicate in Turkish and had no cognitive impairment that would prevent them from communicating, had been diagnosed with RA at least one year ago, were using at least one digital tool (such as a smartphone or computer), and agreed to participate in the study. In line with the study’s objective, individuals using at least one digital tool were included to enable participants to share their experiences. A purposive sampling method was used to identify the individuals who would participate in the study. Two individuals who filled out the data collection tools incompletely/incorrectly and did not complete the interview stage were excluded from the study. The participants were determined using purposive sampling method. Purposive sampling is a sampling method based on the conscious selection of participants who can convey in-depth information on the subject, compatible with the purpose, question, and context of the study [21].

### Data collection process

A “Patient Information Form” [3, 4] and the “Semi-Structured Interview Form” [17, 22–24] were used to collect datam (Appendix 1). These forms, created in accordance with the relevant literature, did not require any external use permission, therefore no permission was requested.

A pilot study was conducted with three patients to evaluate whether the forms were understandable, and these patients were excluded from the primary study. The participants who agreed to participate in the study were informed and their verbal and written consents were obtained. One researcher (KAK) held all interviews in the training room of the outpatient clinic, one-on-one, face-to-face and in the Turkish language. The researcher conducting the interview did not know the participants beforehand and had not been involved in their clinical care processes. The researcher informed the participants that their data would be kept confidential, that they did not work in the relevant that clinical department, and that the interview would not affect their treatment. Each participant was interviewed only once, and a voice recorder was used. Each interview lasted for an average of 40 min (minimum 25 min and maximum 48 min). Data saturation is known to occur when no new code emerges after consecutive interviews. The data was repeated with the 24th patient, and after the interview with the 25th participant, no new code emerged, thus data saturation was reached and the interviews were terminated [25].

### Data analysis

The researcher transcribed the audio recordings within the first 24 h after the interview, including non-verbal expressions (such as laughing, crying, or sighing). The researcher checked the transcribed texts by replaying the audio recordings and then sent the transcripts to the participants for verification of their accuracy. A nickname was used for the participants, such as P1 and P25. The researchers read the transcripts several times and initially coded the data set. The researchers grouped the codes with the same meaning and identified subcategories, categories and themes. The coding was done independently by all researchers and then discussed until a consensus was reached. Interview data were repeatedly reviewed to ensure thematic development. After checking compatibility with the data set, they reviewed and reported the themes they identified [26].

### Ethical considerations

The study was conducted in accordance with the principles of the Helsinki Declaration. Permission was obtained from the relevant institution where the study was conducted (08.05.2025/505708), and approval was obtained from the Hatay Mustafa Kemal University Non-Interventional Clinical Research Ethics Committee (Ethics Approval No. 21.05.2025/23). In addition, verbal and written informed consent was obtained from the participants.

### Reliability

Meetings were held for the reliability of the study, consent from the participants, a detailed presentation of the study design, independent analysis of the data by each researcher, a comparison of the findings with previous studies, and finalisation of the themes. Transferability was achieved by clearly explaining the sample, setting, and process. On the other hand, the confirmability was achieved through a detailed data collection method by considering the views of the researchers and coding the data independently.

### Findings

A total of 25 participants were interviewed in the study. Table 1 shows detailed descriptive characteristics of the participants (Table 1). The data analysis resulted in three main themes and seven sub-themes (Table 2).

**Table 1:** Descriptive characteristics of patients

Variables		Min.-Max.	$\:\stackrel{-}{\boldsymbol{X}}$±SD
Age (years)		18–57	36.4 ± 10.4
DAS28		1.80–3.80	2.76 ± 0.53
Duration of illness (years)		2–30	8.08 ± 6.45
		**n**	**%**
Gender	Women	20	80
	Men	5	20
Marital status	Married	18	72
	Single	7	28
Having children	Yes	13	52
	No	12	48
Education	Primary School	12	48
	High School	8	32
	Undergraduate	4	16
	Postgraduate	1	4
Place of residence	District	21	84
	Village	4	16
Occupation	Freelancer	2	8
	Worker	1	4
	Civil Servant	5	20
	Housewife	17	68
Income	İncome is less than expenses	18	72
	İncome eguals expenses	7	28
Additional illnesses besides RA	Yes	4	16
	No	21	84
Family history of RA	Yes	13	52
	No	12	48
Regular health checkups	Yes	21	84
	No	4	16
Computer/tablet at home	Yes	13	52
	No	12	48

*RA Rheumatoid Arthritis

**DAS28: Disease Activity Score in 28 Joints

**Table 2:** Themes and Sub-themes

Main Themes	Sub-themes
**Main Theme 1**: Use of Digital Health Technologies *Number of sub-themes: 3* *Total kodes: 51* *Total frequency: 365*	**Sub-themes 1**: Used digital health tools *(code number: 12)* **Sub-themes 2**: Intended use *(code number: 15)* **Sub-themes 3**: Researched topics *(code number: 24)*
**Main Theme 2**: Digital health technologies in self-management *Number of sub-themes: 2* *Total kodes: 53* *Total frequency: 199*	**Sub-themes 1**: Positive effects *(code number: 31)* **Sub-themes 2**: Negative effects *(code number: 22)*
**Main Theme 3**: Expectations from digital health technologies *Number of sub-themes: 2* *Total kodes: 48* *Total frequency: 132*	**Sub-themes 1**: Expectations from digital health tools *(code number: 25)* **Sub-themes 2**: Expectations from content *(code number: 23)*

Theme 1: Use of digital health technologies.

This main theme was created based on three sub-themes, i.e., “(1) Used digital health tools,” “(2) Intended use,” and “(3) Researched topics.”

Most of the patients with RA reported that they mostly used Google (22/25) and E-Pulse (22/25) (the national digital health platform), while some of them reported that they used different tools such as mobile health apps, TikTok and artificial intelligence (4/25) (4/25) (9/25).

“…I use artificial intelligence. But I usually use Google; I also check my analyses and results from e-pulse …” (P1).

“I use apps for drinking water and calculating the calories of the food I eat. I also have an app for a pace calculator… Other than that, I have a squad programme.” (P7).

…I’m watching on TikTok, some doctors are showing up… (P3).

Most of the participants frequently used digital health tools to learn about their diseases in detail, to satisfy their curiosities (18/25) and to schedule an appointment (19/25). Some applied to them to find solutions, especially for the periods of exacerbation.

“…I use E-pulse to schedule an appointment…” (P9).

“When I first came across this disease, I researched on Google to find out what it is and how it manifests itself.” (P24).

“When we get sick, we sometimes suffer a lot of pain… Sometimes it happens during sleep. Since we cannot access a medical doctor or go to a hospital at such times, we just search on Google about what to do…” (P22).

The topics most of the participants referred to included disease information (18/25), proper nutrition in RA (17/25), side effects and proper use of drugs (15/25), and different topics such as GETAT methods (9/25).

“I search for what rheumatoid arthritis is. Sometimes I check the ones with video explanations, sometimes the ones with visuals…” (P10).

“I was searching for things that acquaintances recommended; for example, they recommended that turmeric is good for rheumatism… Then I was searching for what is good for rheumatism. What are the side effects of my medication, for example, my injection? Then I learnt about exercise; Pilates is good for me…” (P20).

They also searched whether RA is permanent and what causes it (10/25).

“For example, what is this disease? Is it temporary? What are the effects? I have checked the side effects and stuff like that…” (P4).

Very few patients reported researching different treatment materials (1/25) and serum therapy (biological therapy) in RA (1/25).

“…When I came for my last check-up, the doctor told me I would need IV fluids because my pain hadn’t gone away for a long time. So I got curious and immediately did some research online…” (P25).

…When the pain got really bad, I searched on Google for what I could do and how I could find a solution… Then I bought patches. They worked really well on my wrist, hand, and back…. I bought different creams… (P14).

Theme 2: Digital health technologies in self-management.

This main theme was created according to the sub-themes (1) “Positive effects” and (2) “Negative effects” of participants’ use of DHT in disease self-management.

Some of the patients reported that DHT helped them to manage their diseases effectively (11/25) and facilitate access to disease information (10/25). However, some of them also reported that DHT had positive effects on their self-management, such as learning how to use drugs properly and what to do when side effects appear, how to follow a regular diet, and how to get stronger by exercising.

“…I mean, the research I do on the internet definitely helps me to manage my illness myself… For example, I do pilates regularly… I pay a lot of attention to my diet now. I take my medication regularly…” (P23).

“…For example, I used to stress a lot, well, I learnt how to avoid stress and how to take a walk….” (P25).

“…Since I took cortisone for a long time, I found out that it triggers many diseases. I learnt that it damages internal organs and bones in particular…” (P1).

“…I resumed my treatment due to the information on the internet…” (P6).

“I learnt to be in control… I quit smoking!” (P13).

On the other hand, some of them reported that accessing unfavourable information induced anxiety (9/25) and frightened and upset them (8/25), and they also came across contradictory and misleading information (6/25) in different digital sources.

“What I have learnt from what I have researched, I mean, how can I describe it? It is quite a different situation” (her eyes filled up) … “I mean, a fear builds up in the pain… You go back inside yourself!” You get anxious… You break down psychologically a bit like this…” (P13).

“I would like a straight answer, for example… For example, some sites claim that it might be a temporary disease… I wish there were sites I could trust…” (P4).

“The thing is, you check something, and the medical practitioner tells you something different, Google suggests something different… There is also misinformation…” (P21).

One participant also commented on the ease of use of the exercise and stimulation programs, which had short session durations (1/25).

So, I’m a working woman, I’m very busy, I don’t have much time… I wish there was a short workout… And my phone would constantly give me alerts… It’s easier to do exercises at home with these apps… (P7).

Themes 3: Expectations from digital health technologies.

The third main theme was created based on the sub-themes of (1) “Expectations from digital health tools” and (2) “Expectations from content”.

Some of the participants reported that they expected a wide range of digital health tools to be created, ranging from a site-specific only for RA disease (8/25), a rheumatology physician/nurse hotline service by telephone (5/25), a virtual/digital clock app for lifestyle in RA, to a special app service for elderly patients.

“For example, maybe a programme on this disease only, or something like that! We can access it on the internet… What if everything is explained in detail, such as medicines, treatment, etc…?” (P1).

“If there was a guidance programme… I would like to find experts in the field of rheumatology… When I need to consult about something, for example, I can ask my physician directly online, and I can discuss it. At least I would like someone to be able to get back to me within a certain time…” (P20).

“It can be a virtual clock or a digital clock. It is very useful. Sometimes you put the phone down, it stays on the table… For example, the watch on the arm. The watch should display heartbeat, pulse rate, then pace count, drinking water needs, everything… I mean, it should regulate the lifestyle… Then, when there is an exacerbation… I mean, let’s say the inflammation has intensified… Let the watch signal out something then; for example, it might be a relief…” (P7).

“I wish we were always called on the phone, informed, trained… If they could chat to us like this… I mean, it does not matter whether it is a doctor or a nurse, as long as they contact us…” (P21).

“…A chip that completely cures the disease must be developed…” (P16).

The contents that the participants requested to be included in the digital tools covered many different topics, such as including all disease information specific to RA (8/25), instant access and effect to relieve pain (7), quick access in case of crisis (7), an application suitable for older patients, and even an application that completely cures RA.

“It would treat… Raise awareness…” (P14).

“I wish there was a programme where we could reach the healthcare team at any time… Let’s assume that I have a seizure, I cannot breathe… My report has expired… I have no injection…” (P12).

“‘…for people over 65 years of age who suffer from this disease or who are illiterate, an audible message like an alert, such as ‘Take your medication, take your medication’…” (P8).

“For example, there may be daily instructions from the app… For example, you are a patient with rheumatism. You will consume these foods today. You should do these to avoid the progression of this disease….” (P6).

“…the app could have a messaging program reminding me when to come for my check-up, when my medication runs out, and when my prescription expires…” (P10).

## Discussion

This study utilised in-depth interviews to determine the experiences of individuals with RA in using DHT in their self-management and their expectations from digital health tools.

Individuals with chronic disease have recently demonstrated a significant rise in their tendency to use DHTs [1, 9, 22]. The digital tools that these individuals also apply to range from mobile apps, smartwatches, and activity trackers to internet-based platforms [27]. For example, a systematic review (2024) found that the most frequently used tools in nurse leadership were websites, mobile apps, tracking systems, short text messaging, e-mail and video-based platforms [9]. A study conducted in Germany revealed that approximately half of rheumatology patients used DHT and the most frequently used tools were, respectively, wearable devices, mobile health apps, digital therapies, electronic prescriptions, video calls, and self-blood draws at home [28]. However, the study by Knitza et al., (2020) showed that individuals with chronic rheumatic disease most frequently used the internet in their search for digital health [11]; whereas, another study reported that approximately one-third of RA patients applied to the internet [29]. On the other hand, a qualitative study (2018) indicated that Facebook was the most frequently used platform for patients with RA to seek medical support, whereas Twitter was less preferred [16]. Likewise, a study conducted in Türkiye found that the majority of RA patients resorted to the internet to search for health information [13]. On the other hand, it was observed that almost all patients with RA used the e-Pulse System, a national patient portal (22/25), and the Google search engine (22/25) in the present study. Furthermore, it was determined that the usage rates of digital tools such as mobile health apps, smartwatches, and health websites were low. While the literature shows that digital tools are predominantly used for clinical monitoring, this study primarily utilized social media and internet resources.This result indicates that patients’ use of technically more advanced digital tools that require guidance is limited. This also suggests that the diversity of patients’ use of digital health tools may vary according to their usage level of DHT in the health systems of the countries.

National and international studies have shown that individuals with RA frequently use the internet to learn about their disease [13, 16, 29]. While the present study confirmed that most of the participants used digital tools to search for information (18/25), it also pointed out that their priorities were to schedule an appointment (19/25), unlike the literature. On the other hand, a study conducted in Türkiye showed that most of the patients with RA (62.4%) began to get information on digital media after they were diagnosed, whereas the rate of beginning before and after their diagnosis was the same in the present study [13]. The results showed that RA patients turned to digital health tools in all circumstances and used them even more actively, especially during the course of their illness.

The literature has drawn attention to the broad research of RA in DHT. The topics researched were found to cover a wide range of areas, such as the course of the disease, medications, stress management, follow-up of treatment, alternative therapies, exercise, diet, coping strategies with the disease process, and many more [11, 13, 16, 29, 30]. However, the importance and priority levels given to these topics have varied in all studies, including the present study. Moreover, some individuals in the present study considered DHT as a source of motivation with the intention of accessing information that their disease was non-permanent after diagnosis. This suggests that individuals with RA have recently resorted to DHT on all kinds of issues during the process of uncertainty about the disease and the search for solutions.

It has been reported that DHTs in chronic diseases raise the level of knowledge and awareness and are effective in self-management [7]. Individuals who suffer from rheumatic diseases tend to use DHTs to better understand and manage their diseases [11]. A qualitative study conducted by Mühlensiepen et al., (2021) reported that DHT facilitates access to information, provides time management, and supports self-management in rheumatology patients [31]. A study conducted in Germany revealed that two-thirds of rheumatology patients benefited from DHT [28]. The literature has highlighted self-management as a determinant in effectively coping with RA [1, 5, 32]. A recent study has shown that digital monitoring, including remote assessment tools and wearable devices, is becoming increasingly effective in supporting patient self-management in patients with RA [33]. For example, while a systematic review evaluates the effectiveness of telemedicine interventions in improving self-management in RA patients, our study enriches the literature by comprehensively examining patients’ experiences with DHT in their daily self-management process [18]. Studies on RA patients have shown that, consistent with the current study, DHT facilitates disease management by enabling effective time management [30, 34]. Furthermore, a systematic review indicated that digital tools improved adherence to medication among arthritis patients [35], which also corroborated the present study. Nevertheless, it comes forth as a significant detail that some of the patients (8/25) who discontinued their medication in the present study resumed taking it after completing their digital research. Unlike the literature, another striking finding of the present study was that the participants avoided doing digital research due to their anxiety and fear induced by the possible risks and complications related to the disease they had encountered in the digital media. The findings highlight the importance of healthcare professionals, especially nurses, guiding patients with RA to correct information and psychosocial support in the digital media.

Studies have mainly attributed the effect of DHT on self-management of RA to the reliability of the information. For example, Knitza et al., (2020) found that the lack of knowledge of reliable and scientifically validated apps among rheumatology patients posed a hindrance in the use of mobile health apps [11]. Most of the patients with RA in another study reported that DHT was insufficient for self-management and they had difficulty in determining the accuracy and reliability of the information [16]. Similar to the present study, some patients came across unreliable and conflicting information in the digital environment. However, unlike the study by Morton et al., very few (2/25) of the patients in the present study reported that they followed what they had learned from the digital media without questioning it [7]. Findings of the present study supported the literature by demonstrating that the purpose of using digital health tools by individuals with RA was not only to follow up tests and schedule appointments but also to regulate their lifestyle, to learn about their diseases, and to get support in their self-management.

DHTs allow for the potential to improve the delivery and outcomes of health care for individuals with chronic diseases. However, they can also empower patients and support them individually, resulting in better outcomes than standardised care [11]. A recent scoping review showed that DHT allowed RA patients to take an active role in their care [34]. It can even strengthen the interaction between the patient and the rheumatologist [34] and can be used effectively in communicating with the rheumatology team [7, 11]. Accordingly, a qualitative study indicated that individuals with RA preferred DHT the most in communicating with the rheumatology team [30]. On the other hand, the patients and clinicians in the same study argued that DHT would be more reliable when used under the guidance of a healthcare professional [30]. Another study emphasised that when patients with RA could actively communicate with the healthcare professional, get feedback and feel that they were benefiting, the patients’ willingness to use the digital apps would directly grow [24]. Knudsen et al., (2023) argued that DHTs should be supported by the guidance of healthcare professionals and offered in structures customised to individual needs [17]. However, a systematic review reported that less than half of digital apps offered access to healthcare professionals [12]. The patients in the present study similarly expected digital apps to be designed with the involvement of the rheumatology team, especially the rheumatologist. Also, the remarkable aspect of the present study was that most of the participants had no direct experience of using digital tools. The results show that DHT needs are shaped by unmet needs rather than experience.

The patients with RA in the study by Sia et al., (2025) indicated that DHT was a complementary care and would not substitute face-to-face care [34]. Patients in another study indicated that their primary expectation was to communicate easily and quickly with their rheumatologists, and they mostly used telephone and e-mail to achieve such communication (Knitza et al., [11]. The patients in the present study also expected to seek fast counselling from the physician or nurse via telephone and video conversation. On the other hand, contrary to the result from the literature, no reference was made to counselling via e-mail in the present study. This difference may be attributed to the differences in the place and usage level of DHT in the healthcare services of the countries. Furthermore, even though the international literature has shown that DHT is integrated with the healthcare system, this study showed that it was limited to individual use. This use also suggests that the tools lack the professional and systematic competence to support self-management in RA.

A systematic review revealed that mobile apps specialised for RA patients were insufficient to meet all the needs of patients and offered limited experiences [12]. German patients with rheumatic disease also indicated that mobile health apps alone would be insufficient for self-management [11]. While the literature on RA focuses on assessments of the usability, compliance, and clinical benefit of digital health applications, the present study differs in that it delves deeper into patients’ experiences using their DHT and their perceptions of self-management [15]. The failure of digital apps to deliver a comprehensive experience of RA indicates that the expectations of patients should be set. This study has shown that patients face problems such as difficulty accessing reliable information in the current healthcare system and the lack of healthcare professionals they can consult when needed, especially during flare-ups, and that this situation has increased their expectations from DHT. Studies have shown that the expectations of rheumatology patients from DHT are focused on simple use, reliable information, individual data sources and ease of communication, which corroborates the present study [11, 12]. However, some patients with RA in the present study (8/25) were expecting a solution that would completely eradicate the disease. This suggests that patients’ expectations from DHT are shaped by their perception of the disease and hope for recovery rather than realistic targets for application.

The study by Knudsen et al., (2023) examined patients’ experiences of using a web-based digital educational tool (WebRA) for RA in daily life [17]. While this study brought the evaluations related to digital education into prominence, the present study evaluated the causes of the use of digital health technologies, their impact on self-management and expectations from these tools from a more comprehensive framework. It has been observed that RA patients develop trust in DHT primarily through practices recommended or participated in by healthcare professionals, especially physicians, and through their positive experiences during the disease process. Based on this framework, it highlights the need for reliable and healthcare professional-supported structured systems for the effective use of DST as a self-management tool by patients with RA. It is believed that these structured systems can enhance self-management effectiveness by supporting patient monitoring in clinical practice.

### Limitations

The study has some limitations. First, the use of a qualitative study design and the effects of sampling on selection may limit the generalisability of the findings. Second, Inclusion of non-elderly middle-aged adults and individuals using digital tools in the study may limit the transferability of the findings to older or digitally excluded RA patients. Finally, since the study was conducted at a single centre and within a single country, the findings may not be generalisable to the entire RA population.

## Conclusion

This qualitative study concluded that individuals with RA mainly used DHT for accessing information about the disease, scheduling appointments and supporting self-management in daily life. Additionally, most of the patients indicated that digital health tools integrated with health professionals, especially rheumatology physicians and nurses, should be developed to achieve effective self-management with DHT. This study was not limited to evaluating the use and efficacy of DHT in RA patients. It also contributed to the literature by highlighting patient-centered expectations, challenges, and factors that can guide the digital self-management experience. The findings suggest that DHT is not only related to technological access but also to the health system and policies. Based on the findings, it is recommended to construct digital care models that deliver reliable information under the guidance of nurses and healthcare professionals and are integrated with clinical care processes to strengthen the role of DHT in supporting self-management in RA. It is also recommended that interventional studies with long-term follow-up in different sociocultural scopes should be carried out in this integration.

## Supplementary Information

Below is the link to the electronic supplementary material.

Supplementary Material 1

## Data Availability

The data that support the findings of this study are available from the corresponding author upon reasonable request. The authors declare that the study has been submitted to the journal for the first time, has not been evaluated for publication elsewhere, and has not yet been published elsewhere.
