# Supplementary material for: Experiences of rheumatoid arthritis patients with digital health technologies in self-management: a qualitative study
Source: Rheumatol Int. 2026 Jun 5;46(6):133. doi: 10.1007/s00296-026-06177-2 (PMC13241395; doi:10.1007/s00296-026-06177-2)
Supplement: Supplementary file 1 — Supplementary Material 1 [file 296_2026_6177_MOESM1_ESM.docx]

**Appendix 1. Semi-Structured Interview Form**

| **Code:** ________________ | | **Date:** ___________________ | |
| --- | --- | --- | --- |
| **Start time:** ________________ | | **End time:** ________________ | |
| ***Starting question:*** *Can you briefly introduce yourself?* | | | |
| **Main questions** | **Additional questions** | | **Clarifying questions** |
| **1.** Recently, with the advancement of technology, digital health technologies (applications and platforms accessed via devices such as mobile phones, tablets, and computers) are being used in health-related matters. **Can you describe your experiences using digital health technologies?** | - How did you start using digital health tools?  - Which digital health tools do you use?  - Did you receive support in using digital tools? | | - *Can you expand a little on this?* - *Can you tell me anything else?* - *Can you give me some examples?* |
| **2. How does using digital health technologies affect your disease management?** | - What are the advantages you experience when using digital health tools?  - What are the challenges you experience when using digital health tools? | |  |
| **3. How would you like digital health tools to help you manage your illness more effectively? What are your expectations from these tools?** | - Which digital tools do you think could be more effective?  - How do you think these tools can be made more useful?  - How can healthcare professionals (such as nurses and doctors) support you in this regard?  - How can the use of digital health tools contribute to the effective management of your illness in the future? | |  |
| ***Is there anything else you would like to add or share?*** | | | |
